# Analysis of Mutants Suggests Kamin Blocking in *C. elegans* is Due to Interference with Memory Recall Rather than Storage

**DOI:** 10.1038/s41598-019-38939-3

**Published:** 2019-02-20

**Authors:** Daniel M. Merritt, Justina G. Melkis, Belinda Kwok, Celina Tran, Derek van der Kooy

**Affiliations:** 1 Institute of Medical Science, University of Toronto, Toronto, Ontario, M5S 1A8 Canada; 2 Department of Physiology, University of Toronto, Toronto, Ontario, M5S 1A8 Canada; 3 Department of Molecular Genetics, University of Toronto, Toronto, Ontario, M5S 3E1 Canada

## Abstract

Higher-order conditioning phenomena, including context conditioning and blocking, occur when conditioning to one set of stimuli interacts with conditioning to a second set of stimuli to modulate the strength of the resultant memories. Here we analyze higher-order conditioning in the nematode worm *Caenorhabditis elegans*, demonstrating for the first time the presence of blocking in this animal, and dissociating it from context conditioning. We present an initial genetic dissection of these phenomena in a model benzaldehyde/NH_4_Cl aversive learning system, and suggest that blocking may involve an alteration of memory retrieval rather than storage. These findings offer a fundamentally different explanation for blocking than traditional explanations, and position *C. elegans* as a powerful model organism for the study of higher order conditioning.

## Introduction

Learned associations about the external world are a valuable source of information for animals to exploit in guiding their behavioral choices, but constraints must exist on what associations are learned if they are to remain predictively useful. In rodents, context conditioning and blocking have both been characterized as mechanisms by which memories interact to attenuate or enhance their effects, providing mechanisms by which knowledge about one association can inform the value of others. These “higher-order conditioning” phenomena provide a powerful means where by simple, one-to-one associations about the world can combine in predictively useful ways.

In context conditioning, context cues present during training become associated with a memory such that retrieval of the memory is enhanced in the presence of the context cues, and hindered in alternate contexts. The role of context in associative learning has been extensively studied in rodents and humans (as reviewed by Maren, Phan, & Liberzon^1^, and to a more limited degree in invertebrates^2–4^. Context conditioning has been demonstrated in *Caenorhabditis elegans* for both associative^5^ and non-associative^6^ memories.

In blocking, the learned association between an unconditioned stimulus (US) and a complex conditioned stimulus (CS1 + CS2) is prevented, or “blocked”, if one of the components of the conditioned stimulus is already associated with the US. Blocking is often explained as a failure to attend to the non-associated component of the conditioned stimulus, because the prior association between the associated component and the unconditioned stimulus is sufficient to fully predict the US. In this view, it is necessary that the US be “surprising” to the subject to form new associative memories^7,8^.

Blocking offers some of the strongest evidence that learning depends not on mere temporal contiguity, but on contingency, and is now viewed as an essential phenomenon for any viable theory of learning to explain. The insight that a cue must be “surprising” to be learned about was later expanded and formalized in the influential Rescorla-Wagner model of classical conditioning^9^, which proposed that conditioned stimuli become associated with an unconditioned stimulus based on the scale of the mismatch between the strength of pre-existing associations with the US and the strength of the present association.

Despite blocking having been the subject of extensive investigation in mammals, and having been enormously influential on theories of learning and memory, the neural and molecular mechanisms underlying it remain poorly understood. Studies of blocking in rodents have been plagued by problems of replicability^10^, and researchers have advocated for the adoption of simpler model systems to further our understanding of it^11^.

Efforts to study blocking in invertebrates have met with mixed success. In honeybees (*Apis mellifera*), intramodal blocking has been demonstrated^12^ for training to some odorants. The sea slugs *Hermissenda crassicornis* and *Aplysia californica* both appear to exhibit blocking^2,13^, as does the terrestrial slug *Limax maximus*^14^. Conversely, learning in the fruit fly *Drosophila melanogaster* does not appear to exhibit blocking^15,16^. Blocking in *C. elegans* has not hitherto been shown.

With only 302 neurons in the adult hermaphrodite, each exhibiting relatively determinate development and synaptic connectivity, *C. elegans* offers unique advantages for the analysis of the mechanisms of memory formation, storage and retrieval. Both non-associative forms of learning, including habituation and dishabituation, and associative learning, such as classical conditioning, have been demonstrated in *C. elegans*^17^. More recently, cell-specific molecular readouts of benzaldehyde/food-deprivation memories have been developed for the worm^18^. This well-characterized associative^19^ memory offers a powerful model by which higher order conditioning phenomenon can be analyzed in the worm.

Here we identify slight alterations in training conditions which can selectively reveal both blocking and context conditioning, and provide an initial genetic dissection of their requirements.

## Results

### Optimization of NH_4_Cl learning conditions

**Figure 1:**
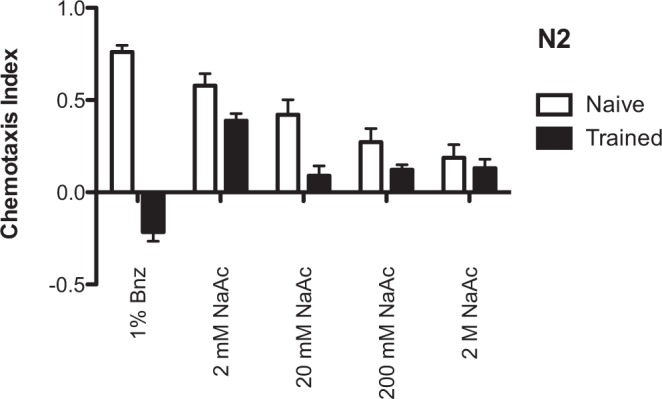
Learning to NH_4_Cl was greatest when tested against a 20 mM point of NaAc. N2 wild type worms were tested for naïve and trained chemotaxis to varying concentrations of NaAc. For salt learning groups, chemotaxis indices are to a 5 μL point of 2.5 M NH_4_Cl opposite a 5 μL point of varying concentrations of NaAc. For the benzaldehyde learning conditions, chemotaxis indices are to a 1 μL point of 1% benzaldehyde opposite a 1 μL point of 100% EtOH. All bars represent mean chemotaxis indices ± SEM after 1 h of food-deprivation on CTX agar (Naïve) or 1 h of food-deprivation on CTX agar with 100 mM NH_4_Cl, except the 1% benzaldehyde group, which represents chemotaxis indices after 1 h of food-deprivation on CTX agar alone (Naïve) or 1 h of food-deprivation on CTX agar with 2 μL 100% benzaldehyde applied to the lid (Trained). N = 9 for all groups except naïve approach to 2 M NaAc, where N = 8.

Before beginning conditioning experiments, we first attempted to determine conditions which would allow for NH_4_Cl/food-deprivation learning equal in extent to benzaldehyde/food-deprivation learning, since previous research has shown that equivalent conditioning strengths are a facilitating condition for blocking^15^. We therefore performed a series of dose response assays to find concentrations of NH_4_Cl and NaAc (CH_3_COONa) that would produce a separation between naïve approach and trained aversion similar in extent to that seen in benzaldehyde learning. We found that training to 100 mM NH_4_Cl, followed by testing on a gradient produced by a 5 μL point of 2.5 M NH_4_Cl opposite a 5 μL point of 20 mM NaAc, produced the greatest alteration of post-training chemotaxis relative to untrained controls, and that which was most similar (albeit not equal in extent) to benzaldehyde/food-deprivation conditioning (Fig. 1). All future blocking experiments using NH_4_Cl conditioning were trained on 100 mM NH_4_Cl plates and tested on plates with opposing gradients of 2.5 M NH_4_Cl and 20 mM NaAc.

### *C. elegans* Memory is Subject to Blocking

**Figure 2:**
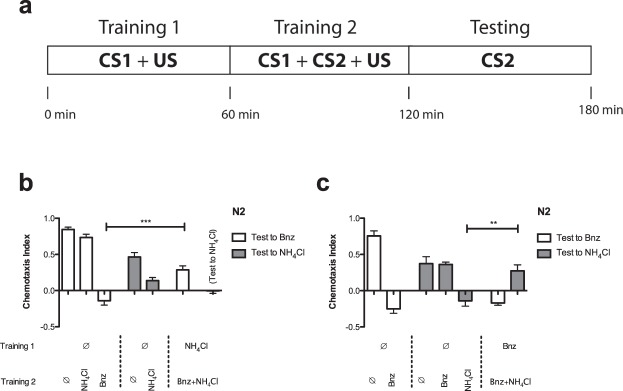
Prior food-deprivation learning to CS1 partially blocks subsequent food-deprivation learning to CS2 in the presence of CS1. (**a**) Time course of the blocking condition. Worms in the blocking condition are first trained for 1 hour to CS1 (NH_4_Cl or benzaldehyde) and the US (food-deprivation), followed by training for one hour to CS1 + CS2 (NH_4_Cl and benzaldehyde) and the US (food-deprivation), before being tested for learning to CS2. (**b**) Prior learning to NH_4_Cl partially blocks benzaldehyde learning. N2 wild-type worms were tested (from left to right) for their approach to benzaldehyde after two hours of food-deprivation alone (no CS, ∅, N = 13), after one hour of food-deprivation followed by one hour exposure to NH_4_Cl (N = 12), and after one hour of food-deprivation followed by one hour exposure to benzaldehyde and food-deprivation (CS2 alone, N = 12), for their approach to NH_4_Cl after two hours exposure to food-deprivation, and after one hour of food-deprivation followed by one hour exposure to food-deprivation and NH_4_Cl (CS1 alone, N = 12), for their approach to benzaldehyde after one hour exposure to food-deprivation and NH_4_Cl followed by one hour exposure to NH_4_Cl, benzaldehyde and food-deprivation (CS1 blocking CS2, diagramed in Fig. 2a, N = 12, Welch’s t = 5.18, df = 22, p = 3.4 × 10^−5^ compared with CS2 alone), and for their approach to NH_4_Cl after one hour exposure to food-deprivation and NH_4_Cl followed by one hour exposure to NH_4_Cl, benzaldehyde and food-deprivation (N = 12). (**c**) Prior learning to benzaldehyde partially blocks NH_4_Cl learning. N2 wild-type worms were tested (from left to right) for their approach to benzaldehyde after two hours of food-deprivation alone (N = 6), after one hour of food-deprivation followed by one hour exposure to benzaldehyde and food-deprivation (CS1 alone, N = 6), for their approach to NH_4_Cl after two hours exposure to food-deprivation (no CS, N = 6), after one hour of food-deprivation followed by one hour exposure to benzaldehyde (N = 6), after one hour of food-deprivation followed by one hour exposure to food-deprivation and NH_4_Cl (CS2 alone, N = 6), for their approach to benzaldehyde after one hour exposure to food-deprivation and benzaldehyde followed by one hour of exposure to food-deprivation, benzaldehyde and NH_4_Cl (N = 6), and for their approach to NH_4_Cl after one hour exposure to food-deprivation and benzaldehyde followed by one hour exposure to NH_4_Cl, benzaldehyde and food-deprivation (CS1 blocking CS2, diagramed in Fig. 2a, N = 6, Welch’s t = 3.72, df = 9, p = 4.8 × 10^−3^ compared to CS2 alone). All bars represent mean chemotaxis indices ± SEM to 1 μL of 1% benzaldehyde (white) or 5 μL of 100 mM NH_4_Cl (grey): **p < 0.01, ***p < 0.001.

To determine whether learning in *C. elegans* exhibits blocking, we starved N2 worms for 1 h in the presence of NH_4_Cl (CS1), before pairing food-deprivation (US) with CS1 and benzaldehyde (CS2) simultaneously for a second hour (Fig. 2a). As expected, worms in naïve control conditions (experiencing only two hours of food-deprivation with no associated CS) underwent strong positive chemotaxis to both NH_4_Cl and benzaldehyde, while those experiencing 1 h of food-deprivation followed by 1 h of food-deprivation paired with NH_4_Cl or benzaldehyde alone subsequently exhibited chemotaxis away from the only the conditioned stimulus. Intriguingly, in the condition in which worms were first trained to NH_4_Cl for an hour before being trained to NH_4_Cl + benzaldehyde for the second hour, although learning to NH_4_Cl (CS1) was similar to that in the CS1-only control condition, learning to benzaldehyde (CS2) was significantly reduced (partially blocked) compared to the CS2-only control condition (Fig. 2b).

In blocking, it is the order of the conditioned stimuli that determines which stimulus is blocked rather than the identities of the stimuli^7^. Thus, we predicted that NH_4_Cl partially blocked benzaldehyde learning because it was presented with the US first, and that the reverse pairing order of benzaldehyde as CS1 and NH_4_Cl as CS2 would give an analogous result in which learning to benzaldehyde blocked learning to NH_4_Cl. As expected, no significant difference was observed between chemotaxis to benzaldehyde (CS1) in this paradigm and in the control, CS1-alone training paradigm, while a significant block of NH_4_Cl (CS2) learning was observed, suggesting that direction of blocking was solely dependent on stimulus order, and not on stimulus identity (Fig. 2c). This dependence on stimulus order further corroborated our identification of this blocking phenomenon with mammalian blocking. To reduce the number of necessary experiments, we focused on the paradigm in which NH_4_Cl (CS1) blocked learning to benzaldehyde (CS2) in subsequent experiments.

### Blocking is Behaviorally Dissociable from Context Conditioning

**Figure 3:**
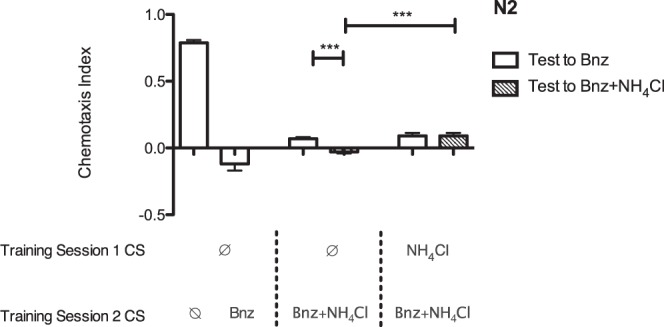
Prior training to CS1 distinguishes context conditioning from blocking. N2 wild-type worms were tested (from left to right) for their approach to benzaldehyde after two hours of food-deprivation alone (no CS, N = 9), after one hour of food-deprivation followed by one hour exposure to benzaldehyde and food-deprivation (CS1 alone, N = 9), for their approach to benzaldehyde after one hour exposure to food-deprivation followed by one hour exposure to benzaldehyde, NH_4_Cl and food-deprivation (CS1 tested without a CS2 context, N = 8), for their approach to benzaldehyde on 100 mM NH_4_Cl CTX after one hour of food-deprivation followed by one hour exposure to food-deprivation, benzaldehyde and NH_4_Cl (CS1 tested with a CS2 context, N = 8, Welch’s t = 5.96, df = 13, p = 4.75 × 10^−5^ compared to CS1 tested without a CS2 context), for their approach to benzaldehyde after 1 hour exposure to food-deprivation and NH_4_Cl, followed by 1 hour exposure to food-deprivation, NH_4_Cl and benzaldehyde (CS1 blocking CS2, N = 9), and for their approach to benzaldehyde on 100 mM NH_4_Cl CTX after 1 hour exposure to food-deprivation and NH_4_Cl, followed by 1 hour exposure to food-deprivation, NH_4_Cl and benzaldehyde (CS1 blocking CS2 with a CS2 context, N = 9, Welch t = 4.59, df = 11, p = 8.0 × 10^−4^ compared to CS1 tested with a CS2 context). All bars represent mean chemotaxis indices ± SEM to 1 μL of 1% benzaldehyde (white), 5 μL of 100 mM NH_4_Cl (grey) or 1% benzaldehyde on 100 mM NH_4_Cl CTX (crosshatched) media: ***p < 0.001.

Training to CS2 in our blocking paradigm occurs in the context of CS1, raising the possibility that the blocking effect we observed resulted not from the order of the training sessions, but because CS2 was learned in a context different from that it was tested in. To distinguish context conditioning from blocking, we tested worms to benzaldehyde (CS2) in the NH_4_Cl (CS1) context they were trained in. We reasoned that if the decreased learning of the CS2/US pairing was the result of context conditioning, matching the testing context to the training context would restore learning, while if it was result of blocking it would not (Fig. 3). We observed that although the presence of CS1 in the testing context restored full learning to CS2 in the absence of a prior CS1-alone training session (context conditioning), failure to learn to CS2 was independent of testing presentation of CS1 in animals that had previously undergone CS1 training (blocking). These results suggested that both context conditioning and blocking could be revealed, were dissociable, and had effects of a similar magnitude.

### Blocking is Independent of Memory Expression to the First Stimulus

**Figure 4:**
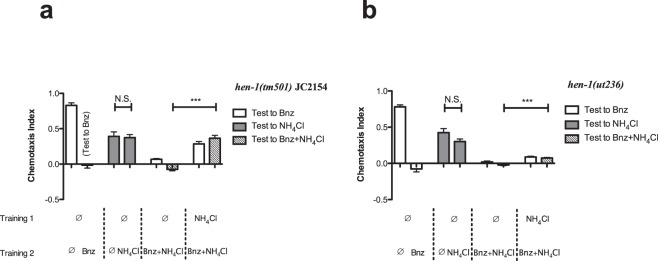
Recall of the CS1/US memory is not required for CS1 to block CS2. (**a**) *hen-1(tm501)* worms were tested (from left to right) for their approach to benzaldehyde after two hours of food-deprivation alone (no CS, N = 8), after one hour of food-deprivation followed by one hour exposure to benzaldehyde and food-deprivation (CS2 alone, N = 8), for their approach to NH_4_Cl after two hours exposure to food-deprivation (no CS, N = 8), and after one hour of food-deprivation followed by one hour exposure to food-deprivation and NH_4_Cl (CS1 alone, N = 8, Welch’s t = 0.33, df = 9, p = 0.75 compared to no CS), for their approach to benzaldehyde after one hour exposure to food-deprivation followed by one hour exposure to NH_4_Cl, benzaldehyde and food-deprivation (N = 8), for their approach to benzaldehyde on 100 mM NH_4_Cl CTX after one hour exposure to food-deprivation followed by one hour exposure to NH_4_Cl, benzaldehyde and food-deprivation (CS2 with a CS1 context, N = 7), for their approach to benzaldehyde after one hour exposure to NH_4_Cl followed by one hour exposure to NH_4_Cl, benzaldehyde and food-deprivation (N = 7), and for their approach to benzaldehyde on 100 mM NH_4_Cl CTX after one hour exposure to NH_4_Cl and food-deprivation followed by one hour exposure to NH_4_Cl, benzaldehyde and food-deprivation (CS1 blocking CS2 with a CS1 context, N = 8, Welch’s t = 7.97, df = 7, p = 4.67 × 10^−5^ after Bonferroni correction compared to CS2 with a CS1 context). (**b**) *hen-1(ut236)* worms were tested as in Fig. 4a (all N = 9). For CS1 alone compared to no CS, Welch’s t = 1.86, df = 13, p = 0.085. For CS1 blocking CS2 with a CS1 context, Welch’s t = 8.03, df = 15, p = 4.11 × 10^−7^ after Bonferroni correction compared to CS2 with a CS1 context. All bars represent mean chemotaxis indices ± SEM to 1 μL of 1% benzaldehyde (white), 5 μL of 100 mM NH_4_Cl (grey) or 1% benzaldehyde on 100 mM NH_4_Cl CTX (crosshatched) media: ***p < 0.001; N.S. p ≥ 0.05.

To further identify where in the psychological learning pathway blocking occurs, we used the well-studied salt learning mutant *hen-1(tm501)*. *hen-1* encodes a secreted protein expressed in ASE neurons whose activity is required for the integration of sensory stimuli and behavioral plasticity^20,21^. While it has previously been reported that *hen-1(tm501)* mutations result in a partial loss of NaCl/food-deprivation associative learning, we find that under our training conditions *hen-1(tm501)* fully eliminates learning to NH_4_Cl, while preserving salt sensation. We predicted that *hen-1(tm501)*, being unable to pair NH_4_Cl (CS1) to the US, will be able to fully pair benzaldehyde (CS2) to the US, and thus exhibit no blocking of benzaldehyde learning. When tested to benzaldehyde in the blocking condition, however, *hen-1(tm501)* surprisingly showed a partial block of benzaldehyde learning similar to that of N2 (Fig. 4a). This block of learning could not be restored by matching testing context to the CS2 training context, suggesting that blocking, and not context conditioning, was responsible. Worms homozygous for *hen-1(ut236)* – an independent loss of function *hen-1* allele^21^ – showed a similar set of phenotypes (Fig. 4b). These data, revealing that a mutation can eliminate learning to CS1 without hindering blocking by CS1, suggest that blocking may be occurring independent of learning to CS1.

To further analyze the genetic requirements for blocking, we investigated the requirement for the CREB ortholog *crh-1* in blocking. *crh-1(tz2)* worms homozygous for a putative null allele of CREB1 exhibited wild type learning and blocking in a NH_4_Cl blocking benzaldehyde paradigm (Supplemental Fig. 1). This is consistent with previous reports that *crh-1* is necessary for spaced but not massed training^22^.

### Blocking Does Not Prevent EGL-4 nuclear localization in AWC

We next utilized the strain JZ500, containing an EGL-4::GFP fusion protein, to position the observed blocking of benzaldehyde learning within the known benzaldehyde learning pathway. EGL-4 is a Protein Kinase G ortholog. The cytoplasmic to nuclear translocation of EGL-4 within the benzaldehyde sensing AWC neuron is required for benzaldehyde/food-deprivation learning^18,23^. We reasoned that if the observed blocking occurs upstream of EGL-4 nuclear localization in AWC, JZ500 worms in the blocking condition should exhibit EGL-4 nuclear localization similar to benzaldehyde/food-deprivation naive animals, while if it occurs downstream localization of this protein should be similar to benzaldehyde/food-deprivation trained animals.

**Figure 5:**
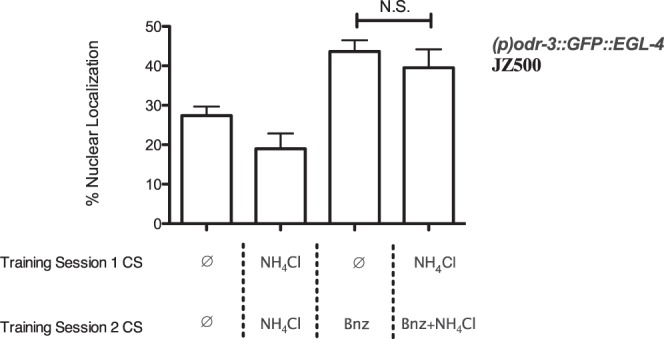
Nuclear localization of EGL-4::GFP occurs even during blocking of the benzaldehyde/food-deprivation memory. JZ500 (*pyIs500 [ofm-1p::GFP* + *odr-1p::dsRed* + *odr-3p::GFP::egl-4]*) worms were trained (left to right) for two hours to food-deprivation alone (N = 8), for two hours to NH_4_Cl and food-deprivation (N = 9), for one hour of food-deprivation followed by one hour training to benzaldehyde and food-deprivation (CS2 alone, N = 9), and for one hour of food-deprivation and NH_4_Cl followed by one hour of food-deprivation, NH_4_Cl and benzaldehyde (CS1 blocking CS2, N = 8, Welch’s t-test t = 0.75, df = 11, p = 0.47 compared to CS2 alone). All bars represent percent nuclear localized EGL-4::GFP: N.S. p ≥ 0.05. JZ500 exhibited behavioural blocking similar to N2 (Supplemental Fig. 2).

Naive worms and worms conditioned to NH_4_Cl alone showed small and statistically indistinguishable EGL-4 nuclear localization, while worms trained in the NH_4_Cl blocking benzaldehyde paradigm showed greater and similar EGL-4 nuclear localization to worms conditioned to food-deprivation and benzaldehyde alone, suggesting that the mechanism of the observed blocking effect is downstream of EGL-4 nuclear localization (Fig. 5). JZ500 worms exhibited blocking behaviorally similarly to N2, confirming that the observed EGL-4::GFP nuclear localization was not caused by a failure to undergo blocking (Supplemental Fig. 2). This finding suggests that blocking of the CS2/US association may occur after formation of the CS2 memory, during recall.

## Discussion

### Blocking and Context Conditioning are Distinguished by Prior Conditioning

We find that both blocking and context conditioning occur in *C. elegans*, and that either can be displayed given slight alterations to a model NH_4_Cl/benzaldehyde learning paradigm. Prior exposure to a first conditioned stimulus and the unconditioned stimulus is both necessary and sufficient to result in blocking during subsequent conditioning to both conditioned stimuli and the unconditioned stimulus. In the absence of prior exposure to CS1, context conditioning occurs, with an effect on learning to CS2 that can be obscured or revealed by adding or removing CS1 from the testing context, respectively.

Previous work has reported several paradigms under which context conditioning occurs in *C. elegans*, but this is the first report of blocking in the species. While the extent of blocking varied between our experiments, a statistically significant effect was reliably observed. Notably, blocking in *C. elegans* appears to be of a more limited magnitude than in rodents: while blocking in rodents often results in a complete elimination of learning to CS2, our best conditions only result in a partial reduction of learning to CS2. This may reflect non-optimal parameters in our blocking paradigm, or an independent (and more limited) evolution of blocking in *C. elegans* than in mammalian brains.

### Blocking of Benzaldehyde Learning May Occur Downstream of EGL-4 Nuclear Localization

In principle, blocking could exert its effect either before on (or before) memory formation, or on memory retrieval. Nuclear localization of EGL-4 in AWC has been shown to be both necessary and sufficient for benzaldehyde/food-deprivation associative learning^18^. We find that nuclear localization of EGL-4 in AWC occurs even when the behavioral output of benzaldehyde/food-deprivation learning has been partially blocked by prior NH_4_Cl learning. This is consistent with blocking of benzaldehyde learning occurring downstream or parallel to EGL-4 nuclear localization in AWC, perhaps in a pathway involved in memory retrieval. Since benzaldehyde is sensed by the AWC neurons^24^, while NH_4_Cl is primarily sensed in an AWC-independent fashion^25^, blocking may be occurring in a downstream interneuron.

### Blocking Occurs Independent of Learning to CS1

Our finding that *hen-1(tm501)* worms, which are incapable of NH_4_Cl/food-deprivation associative learning, can still undergo blocking, suggests that learning to CS1 is not required for CS1 to block CS2. Since CS1 would not seem to have any predictive value for the unconditioned stimulus in these animals but remains capable of blocking learning to CS2, this finding appears to challenge the Rescorla-Wagner model of learning, which hypothesizes that blocking of a CS occurs when CS1 alone is sufficient to predict the US.

A possible mechanism by which HEN-1 could be required for NH_4_Cl learning, but not for NH_4_Cl blocking of benzaldehyde learning, is that HEN-1 is required for recall of the NH_4_Cl associative memory, but not for its formation, and that the presence of the memory alone (without the ability to alter chemotactic behavior based on it) is sufficient to result in blocking of the benzaldehyde associative memory.

If EGL-4 nuclear localization is close to the formation of the benzaldehyde/food-deprivation engram, our finding that this occurs normally in salt blocked wild type worms suggests that salt blocking may not involve hindered storage of the benzaldehyde memory, but rather interference of salt to the retrieval of the benzaldehyde memory. Moreover, this interference must be specific to the mechanisms of blocking and not context conditioning, where salt appears to enhance the retrieval of the context memory. Inhibition of recall of an association during blocking, rather than inhibition of formation of the association, is similarly inconsistent with the predictions of the Rescorla-Wagner model.

A mechanism for blocking based on retrieval-failure has previously been proposed in rats^26^. That retrieval-failure is similarly the most parsimonious explanation of blocking in *C. elegans* both strengthens evidence for it as a broadly cross-species mechanism of higher level learning, and demonstrates the utility of *C. elegans* as a genetically tractable model in which to infer the mechanisms of higher order mammalian learning.

## Materials and Methods

### Strains

Wild-type N2 (Bristol strain), *hen-1(tm501)* JC2154, *pyIs500 [ofm-1p::GFP* + *odr-1p::dsRed* + *odr-3p::GFP::egl-4]* JZ500 were obtained from the Caenorhabditis Genetics Center (CGC), University of Minnesota, which is funded by NIH Office of Research Infrastructure Programs (P40 OD010440). *hen-1(ut236)* was a generous gift from Takeshi Ishihara.

### Cultivation and General Methods

Animals were cultivated on nematode growth medium (NGM) seeded with OP50 *Escherichia coli*^27^. Age-synchronized worms were grown at 23 °C until the young adult stage (48 h) before behavioral experiments. All behavioral assays were performed on 100 mm chemotaxis media (CTX) petri plates^5^ at 20 °C. Behavioral experiments were performed on In assays testing the approach to salts, salt gradients were established by adding 5 μL of the indicated concentration of ammonium chloride (NH_4_Cl) or sodium acetate (NaAc) approximately 3.5 h before testing. In assays testing benzaldehyde (Bnz) approach, 1 μL 1% benzaldehyde and 1 μL 100% ethanol (EtOH) were added to opposite sides of the CTX plate 2 cm from the edges immediately after worms were placed on the plate during testing. Immediately following, 1 μL of 1 M sodium azide was placed on top of each point of benzaldehyde, ethanol, NH_4_Cl and NaAc to anaesthetize the worms, preserving their initial choice. Before beginning the assays, worms were washed from the cultivation plates and rinsed twice with sterile water to remove residual bacteria. Milli-Q water was used in every transfer to another plate before worms were dried with Kimwipes. All chemicals were purchased from Sigma-Aldrich, St. Louis, Missouri. All testing phases allowed worms to undergo chemotaxis for 1 h, after which they were counted. Dead or damaged worms were not scored. The Chemotaxis Index (CI) was calculated according to the equation CI = ((# worms at salt/odorant point) - (# worms at counterpoint))/(# worms on plate). Worms were scored as being at a point if they were within a 20 mm radius of the point of salt or odorant placement. A score of +1 indicates complete approach to either NH_4_Cl or benzaldehyde, while a score of −1 indicates complete avoidance. For behavioural assays, all groups in single graphs were run on the same day under the same conditions.

### Experimental Design and Statistical Analysis

For experiments where multiple tests were performed, p-values were adjusted using Bonferroni corrections. All assays were run on at least two days, in duplicate or triplicate, with 20–400 young adult worms per plate. Plates having fewer than 20 or greater than 400 worms were excluded from analysis. As this sometimes resulted in uneven sample sizes between conditions, all comparisons for statistical significance were made with Welch’s variant of two-sided t-tests. All Ns represent number of plates, except in Fig. 5, where N represents number of animals.

### Dose Response Salt Assays

5 μL of 2.5 M NH_4_Cl was used to establish a salt gradient during testing against an increasing NaAc gradient (2 mM to 2.5 M), as indicated. For all salt chemotaxis experiments, gradients were allowed to develop in the agar for 3–5 hours before testing. For naïve approach assays, worms were placed on CTX and allowed to chemotax for 1 h to the previously established salt gradients. For NH_4_Cl learning assays, worms were starved for 1 h on 100 mM NH_4_Cl CTX agar before being transferred to CTX testing plates with prior established salt gradients, and allowed to chemotax for 1 h.

### Blocking assays

#### NH_4_Cl blocking Benzaldehyde

In the blocking condition, worms were adapted to 100 mM NH_4_Cl for 1 h, prior to the addition of 2 μL of 100% benzaldehyde on the lid for a second hour, before being tested to salt (2.5 M NH_4_Cl vs 20 mM NaAc) or benzaldehyde (1 μL of 1% benzaldehyde in EtOH vs 1 μL 100% EtOH) separately. In the naïve condition, worms were left to starve for 2 h and tested to NH_4_Cl or benzaldehyde. In the benzaldehyde trained control, worms were starved for 1 h before being transferred to new plates to which 2 μL 100% benzaldehyde was added to the lid, after which they were conditioned for 1 h before testing. In the NH_4_Cl trained control, worms were starved for 1 h before being transferred to 100 mM NH_4_Cl CTX plates and starved for 1 h before testing.

#### Benzaldehyde blocking NH_4_Cl

Worms were adapted to 2 μL of 100% benzaldehyde for 1 h prior to exposure to both NH_4_Cl and benzaldehyde for 1 h. The naïve condition was performed identically to the NH_4_Cl blocking benzaldehyde paradigm. Worms were starved in the presence of 2 μL 100% benzaldehyde in the benzaldehyde trained control. They were starved for 1 h before being transferred to 100 mM NH_4_Cl CTX plates for 1 h in the NH_4_Cl trained control.

#### Blocking vs Context conditioning

Performed identically to the NH_4_Cl blocking Benzaldehyde assay, except worms in the blocking condition were also tested for benzaldehyde approach on 100 mM NH_4_Cl CTX agar.

### Confocal microscopy

Worms were conditioned in the NH_4_Cl blocking benzaldehyde paradigm with naïve, and NH_4_Cl and benzaldehyde trained controls as described above. Animals were washed from the training plates and placed on an agarose pad on a slide, paralyzed with 1 mM levamisole, and covered with a cover slip. Slides were imaged immediately on an Olympus Fluoview FV1000 confocal microscope using a 60x oil immersion objective and analyzed with ImageJ. Percent GFP nuclear localization was calculated as the fraction of GFP found in the cell nucleus divided by the total present in the cell.

## Supplementary information

Supplementary Figures

## Data Availability

All datasets generated in this study are available from the authors upon request.

## References

[1] Maren S, Phan KL, Liberzon I (2013). The contextual brain: implications for fear conditioning, extinction and psychopathology. Nat. Rev. Neurosci..

[2] Colwill RM, Absher RA, Roberts ML (1988). Context-US learning in Aplysia californica. J. Neurosci..

[3] Brembs B, Wiener J (2006). Context and occasion setting in *Drosophila* visual learning. Learn. Mem..

[4] Gerber B, Menzel R (2000). Contextual Modulation of Memory Consolidation. Learn. Mem..

[5] Law E, Nuttley WM, van der Kooy D (2004). Contextual taste cues modulate olfactory learning in *C. elegans* by an occasion-setting mechanism. Curr. Biol..

[6] Rankin CH (2000). Context conditioning in habituation in the nematode *Caenorhabditis elegans*. Behav. Neurosci..

[7] Kamin, L. J. In *Punishment and aversive behavior* 279–296, 10.2105/AJPH.2006.090910 (1969).

[8] Kamin, L. J. ‘Attention-like’ processes in classical conditioning. In *Miami symposium on the prediction of behavior, 1967: Aversive stimulation* (ed. Jones, M. R.) 9–31 (University of Miami Press, 1968).

[9] Rescorla, R. A. & Wagner, A. R. In *Classical conditioning II: current research and theory* (eds Black, H. A. & Prokasy, W. F.) 64–99 (Appleton-Century-Crofts, 1972).

[10] Maes, E. *et al*. The elusive nature of the blocking effect: 15 failures to replicate. *J. Exp. Psychol. Gen*. (2016). 10.1037/xge0000200 27428670

[11] Bitterman ME (2006). Classical conditioning since Pavlov. Rev. Gen. Psychol..

[12] Couvillon PA, Arakaki L, Bitterman ME (1997). Intramodal blocking in honeybees. Anim. Learn. Behav..

[13] Rogers RF, Matzel LD (1996). Higher-order associative processing in *Hermissenda* suggests multiple sites of neuronal modulation. Learn. Mem..

[14] Sahley, C., Rudy, J. W. & Gelperin, A. An analysis of associative learning in a terrestrial mollusc. I. Higher-order conditioning, blocking, and a transient US preexposure effect. *J. Comp. Physiol*. 1–8 (1981).

[15] Brembs B, Heisenberg M (2001). Conditioning with compound stimuli in *Drosophila melanogaster* in the flight simulator. J. Exp. Biol..

[16] Young JM, Wessnitzer J, Armstrong JD, Webb B (2011). Elemental and non-elemental olfactory learning in *Drosophila*. Neurobiol. Learn. Mem..

[17] Rankin, C. H. Invertebrate learning: What can’t a worm learn? *Current Biology* **14** (2004). 10.1016/j.cub.2004.07.044 15296777

[18] Lee JI (2010). Nuclear entry of a cGMP-dependent kinase converts transient into long-lasting olfactory adaptation. Proc. Natl. Acad. Sci. USA.

[19] Nuttley WM, Atkinson-Leadbeater KP, van der Kooy D (2002). Serotonin mediates food-odor associative learning in the nematode Caenorhabditis elegans. Proc. Natl. Acad. Sci. USA.

[20] Altun-Gultekin Z (2001). A regulatory cascade of three homeobox genes, *ceh-10*, *ttx-3* and *ceh-23*, controls cell fate specification of a defined interneuron class in *C. elegans*. Development.

[21] Ishihara T (2002). HEN-1, a secretory protein with an LDL receptor motif, regulates sensory integration and learning in *Caenorhabditis elegans*. Cell.

[22] Kauffman AL, Ashraf JM, Corces-Zimmerman MR, Landis JN, Murphy CT (2010). Insulin signaling and dietary restriction differentially influence the decline of learning and memory with age. PLoS Biol..

[23] O’Halloran, D. M., Altshuler-Keylin, S., Lee, J. I. & L’Etoile, N. D. Regulators of AWC-mediated olfactory plasticity in *Caenorhabditis elegans*. *PLoS Genet*. **5**, (2009). 10.1371/journal.pgen.1000761 PMC2780698 20011101

[24] Bargmann CI, Hartwieg E, Horvitz HR (1993). Odorant-selective genes and neurons mediate olfaction in *C. elegans*. Cell.

[25] Frøkjær-Jensen, C., Ailion, M. & Lockery, S. R. Ammonium-acetate is sensed by gustatory and olfactory neurons in Caenorhabditis elegans. *PLoS One* **3** (2008). 10.1371/journal.pone.0002467 PMC2413426 18560547

[26] Balaz MA, Gutsin P, Cacheiro H, Miller RR (1982). Blocking as a retrieval failure: Reactivation of associations to a blocked stimulus. Q. J. Exp. Psychol..

[27] Brenner S (1974). The genetics of *Caenorhabditis elegans*. Genetics.

