# Supplementary material for: Analysis of Mutants Suggests Kamin Blocking in C. elegans is Due to Interference with Memory Recall Rather than Storage
Source: Sci Rep. 2019 Feb 20;9:2371. doi: 10.1038/s41598-019-38939-3 (PMC6382802; doi:10.1038/s41598-019-38939-3)
Supplement: Supplementary file 1 — Supplementary Figures [file 41598_2019_38939_MOESM1_ESM.docx]

Analysis of Mutants Suggests Kamin Blocking in 
*C. elegans* is Due to Interference with Memory Recall
 Rather than Storage

Daniel M. Merritt, Justina G. Melkis, Belinda Kwok, Celina Tran & Derek van der Kooy

## Supplemental Figures

**Supplemental Figure 1: *crh-1* is not required for blocking or for benzaldehyde or NH_4_Cl learning.** *crh-1(tz2)* worms were tested (from left to right) for their approach to benzaldehyde after two hours of starvation alone (no CS, N=9), after one hour of starvation followed by one hour exposure to benzaldehyde and starvation (CS1 alone, N=9), for their approach to NH_4_Cl after two hours exposure to starvation (N=9 plates), and after one hour of starvation followed by one hour exposure to starvation and NH_4_Cl (CS1 alone, N=9 plates), for their approach to benzaldehyde after one hour exposure to starvation followed by one hour exposure to NH_4_Cl, benzaldehyde and starvation (N=9 plates), for their approach to benzaldehyde on 100 mM NH_4_Cl CTX after one hour exposure to starvation followed by one hour exposure to NH_4_Cl, benzaldehyde and starvation (N=9 plates), for their approach to benzaldehyde after one hour exposure to NH_4_Cl followed by one hour exposure to NH_4_Cl, benzaldehyde and starvation (CS1 blocking CS2, N=9 plates, Welch’s t=5.65, df=16, p=3.61x10^-5^ compared to CS2 alone), and for their approach to benzaldehyde on 100 mM NH_4_Cl CTX after one hour exposure to NH_4_Cl and starvation followed by one hour exposure to NH_4_Cl, benzaldehyde and starvation (N=9 plates). All bars represent mean chemotaxis indices ± SEM to 1 μL of 1% benzaldehyde (white), 5 μL of 100 mM NH_4_Cl (grey) or 1% benzaldehyde on 100 mM NH_4_Cl CTX (crosshatched) media; Welch’s two-tailed t test was used to determine significance: *** p < 0.001.

**Supplemental Figure 2: JZ500 exhibits behavioral blocking.** JZ500 (*pyIs500 [ofm-1p::GFP + odr-1p::dsRed + odr-3p::GFP::egl-4]*) worms were tested (from left to right) for their approach to benzaldehyde after two hours of starvation alone (no CS, N=9 plates), after one hour of starvation followed by one hour exposure to benzaldehyde and starvation (CS2 alone, N=9 plates), for their approach to NH_4_Cl after two hours exposure to starvation (N=9 plates), and after one hour of starvation followed by one hour exposure to starvation and NH_4_Cl (CS1 alone, N=9 plates), for their approach to benzaldehyde after one hour exposure to starvation followed by one hour exposure to NH_4_Cl, benzaldehyde and starvation (N=9 plates), for their approach to benzaldehyde on 100 mM NH_4_Cl CTX after one hour exposure to starvation followed by one hour exposure to NH_4_Cl, benzaldehyde and starvation (N=9 plates), for their approach to benzaldehyde after one hour exposure to NH_4_Cl followed by one hour exposure to NH_4_Cl, benzaldehyde and starvation (CS1 blocking CS2, N=9 plates, Welch’s t=5.8, df=13, p=6.17x10^-5^ compared to CS2 alone), and for their approach to benzaldehyde on 100 mM NH_4_Cl CTX after one hour exposure to NH_4_Cl and starvation followed by one hour exposure to NH_4_Cl, benzaldehyde and starvation (N=9 plates). All bars represent mean chemotaxis indices ± SEM to 1 μL of 1% benzaldehyde (white), 5 μL of 100 mM NH_4_Cl (grey) or 1% benzaldehyde on 100 mM NH_4_Cl CTX (crosshatched) media; Welch’s two-tailed t test was used to determine significance: *** p < 0.001.
